# Comparison of mortality and clinical failure rates between vancomycin and teicoplanin in patients with methicillin-resistant *Staphylococcus aureus* pneumonia

**DOI:** 10.1186/s12879-022-07549-2

**Published:** 2022-07-07

**Authors:** Jang Ho Lee, Myeong Geun Choi, Hyung Jun Park, Ho Cheol Kim, Chang-Min Choi

**Affiliations:** 1 Department of Pulmonology and Critical Care Medicine, Asan Medical Centre, University of Ulsan College of Medicine, Seoul, Republic of Korea; 2 Department of Oncology, Asan Medical Center, University of Ulsan College of Medicine, Seoul, Republic of Korea; 3 Division of Pulmonology and Critical Care Medicine, Department of Internal Medicine, Asan Medical Centre, University of Ulsan College of Medicine, 88 Olympic-ro 43-gil, Songpa-gu, Seoul, 05505 Republic of Korea

**Keywords:** Treatment, Vancomycin, Teicoplanin, Pneumonia, Methicillin-resistant *Staphylococcus aureus*

## Abstract

**Background:**

Very few studies have compared the effects and side effects of vancomycin and teicoplanin in patients with methicillin-resistant *Staphylococcus aureus* pneumonia. This study aimed to compare the efficacy and safety of vancomycin and teicoplanin in patients with methicillin-resistant *Staphylococcus aureus* pneumonia.

**Methods:**

This study examined 116 patients with methicillin-resistant *Staphylococcus aureus* pneumonia who met the inclusion criteria and were treated with either vancomycin (*n* = 54) or teicoplanin (*n* = 62). The primary (i.e., clinical failure during treatment) and secondary outcomes (i.e., mortality rates, discontinuation of study drugs due to treatment failure, side effects, and clinical cure) were evaluated.

**Results:**

The vancomycin group presented lower clinical failure rates (25.9% vs. 61.3%, *p* < 0.001), discontinuation due to treatment failure (22.2% vs. 41.9%, *p* = 0.024), and mortality rates (3.7% vs 19.4%, *p* = 0.010). The Cox proportional hazard model revealed that teicoplanin was a significant clinical failure predictor compared with vancomycin (adjusted odds ratio, 2.198; 95% confidence interval 1.163–4.154). The rates of drug change due to side effects were higher in the vancomycin group than in the teicoplanin group (24.1% vs. 1.6%, *p* < 0.001).

**Conclusions:**

Vancomycin presented favorable treatment outcomes and more side effects compared with teicoplanin, which suggests that clinicians would need to consider the efficacy and potential side effects of these drugs before prescription.

**Supplementary Information:**

The online version contains supplementary material available at 10.1186/s12879-022-07549-2.

## Background

Methicillin-resistant *Staphylococcus aureus* (MRSA) is one of the most common and lethal pathogens [1, 2]. Although MRSA can cause several infectious diseases in various organs of the human body, it is considered an important causative agent of pneumonia [2–4]. The morbidity and mortality rates of pneumonia caused by MRSA are higher than those caused by other causative pathogens [5]. Therefore, proper early treatment is vital for clinicians and patients with MRSA pneumonia.

Several clinical guidelines recommended vancomycin as one of the first-line treatments for MRSA pneumonia [6]. Nephrotoxicity is well-known adverse effects of vancomycin. Severe MRSA infections often cause nephrotoxicity, which may be exacerbated by the use of certain antimicrobial agents, such as vancomycin, in inappropriate doses [7]. Therefore, clinicians should use vancomycin with caution in patients with increased risk of side effects such as azotemia [8]. If the adverse effects of vancomycin were worrying in certain situations, especially nephrotoxicity, teicoplanin was regarded as a useful alternative of vancomycin in several MRSA infections [9]. Moreover, teicoplanin had a relatively lower rate of adverse effects than vancomycin, ease of administration, and once-daily regimen due to a long half-life [10, 11]. Although teicoplanin is not actively recommended for the treatment of MRSA pneumonia, some clinicians use teicoplanin for patients with MRSA pneumonia in clinical practice [9, 12]. However, this practice is controversial due to a lack of evidence.

Vancomycin and teicoplanin are included in glycopeptide antibiotics and disrupt cell wall synthesis in gram-positive bacteria by obstructing peptidoglycan biosynthesis [13, 14]. Many studies insist that there is no difference in the efficacy between vancomycin and teicoplanin in MRSA infection [10, 15–17]. However, in a previous study, these drugs presented different clinical features, including the type of adverse event, plasma albumin binding rate, and tissue penetration [18]. Therefore, further studies will be required to compare the efficacy and safety of vancomycin and teicoplanin for MRSA pneumonia because the lung penetration of glycopeptide antibiotics was found to be relatively low [13].

This study aimed to compare the efficacy and safety of vancomycin and teicoplanin. To achieve this, the clinical failure rate, including mortality and change of the study drugs to other drugs due to treatment failure, between vancomycin and teicoplanin were evaluated. Additionally, the mortality rate, treatment failure, side effects, and clinical cure were compared between the study drugs.

## Methods

### Study design and participants

A single-center retrospective study was conducted at Asan Medical Center, which is a 2700-bed referral hospital in Seoul, South Korea. Eligible patients (age ≥ 18 years) were selected based on the prescription of vancomycin or teicoplanin for MRSA pneumonia from 2015 to 2019 in the electronic medical record system. The inclusion criteria were as follows: (1) MRSA identified from sputum and/or blood cultures, (2) radiological evidence of pneumonia, (3) clinical diagnosis of MRSA pneumonia based on the clinicians’ judgments, (4) no other microorganisms recognized as potential causative agents of MRSA, and (5) selection of vancomycin or teicoplanin by clinicians as the first antibiotic for MRSA pneumonia. The exclusion criteria were as follows: (1) patients were transferred to other hospitals before treatment completion, (2) the study drugs were initiated at other hospitals, (3) patients with extracorporeal membrane oxygenation, (4) MRSA was isolated only in inadequate sputum samples and not in adequate sputum and blood cultures, and (5) patients had < 1 month of life expectancy based on underlying diseases and judgment of the attending physicians.

This study protocol was approved by the Institutional Review Board of Asan Medical Center (IRB No.: 2020-1667). The board waived the request for informed consent due to the retrospective nature of this study. All patient data were anonymized.

### Vancomycin and teicoplanin administration

Because there was no institutional guideline for selecting vancomycin or teicoplanin for MRSA infection at our hospital, the choice of study drugs was determined by the physicians’ judgment or preference, considering risk factors for vancomycin nephrotoxicity, such as baseline serum creatinine level, underlying renal disease, and pneumonia severity. Vancomycin (15 mg/kg every 12 h) was prescribed at first, and the dose of the drugs was changed targeting trough levels of 15–20 µg/mL. The clinicians periodically investigated the serum trough levels of vancomycin to adjust the vancomycin dose. In the teicoplanin group, the patients received three teicoplanin loading doses of 12 mg/kg every 12 h, followed by maintenance doses of 12 mg/kg in the high-dose regimen. In the low-dose regimen, patients received three teicoplanin loading doses of 6 mg/kg every 12 h, followed by maintenance doses of 6 mg/kg. The physicians selected the teicoplanin regimen based on their clinical judgments. The frequency of maintenance doses was determined based on creatinine clearance. The teicoplanin maintenance dose was administered daily, every 48 h, and every 72 h in patients with creatinine clearances of > 80, 30–80, and 30 mL/min, respectively.

### Measurements

The primary outcome of this study was the clinical failure rates, including mortality and change of the first drug to other drugs because of treatment failure. The secondary outcomes included mortality rate, change of the study drug due to treatment failure, discontinuation of the study drug due to side effects, and clinical cure. All outcomes were analyzed during the therapeutic period with the initial study drugs. Treatment failure or side effects were annotated based on the combination of the clinicians’ judgment and change to other drugs for further MRSA pneumonia treatment. We investigated all-cause mortality during hospitalization and the periods with study drugs or other anti-MRSA drugs, which were changed due to treatment failure of the study drugs. Clinical cure was defined when the clinicians determined that MRSA pneumonia was resolved based on the patients’ symptoms, improvement of chest X-ray findings, and laboratory findings and that the patients did not need to take additional antibiotics for MRSA pneumonia treatment. We investigated the occurrence of adverse events during the therapeutic period, which led to the discontinuation of the study drugs. Azotemia was defined as any event of at least two consecutive measurements of increase by ≥ 0.5 mg/dL or 50% above the baseline serum creatinine level [19].

The baseline characteristics and comorbidities were analyzed in both groups. The types of pneumonia were divided into three categories: community-acquired pneumonia (CAP), hospital-acquired pneumonia (HAP), and ventilator-associated pneumonia (VAP) [20, 21]. HAP and VAP were defined as pneumonia that occurred ≥ 48 h after admission and > 48 h after endotracheal intubation, respectively [20]. Other pneumonia cases were classified as CAP. We investigated the coexisting conditions based on the electronic medical records and associated drugs use. If the combined disease was not cured before the start date of vancomycin or teicoplanin administration and the associated drugs were administered for the combined disease, we considered it as coexisting conditions. The history of recent chemotherapy and radiotherapy was investigated within 3 months before the start date of vancomycin or teicoplanin administration. Laboratory findings, vital sign data, and the Acute Physiology and Chronic Health Enquiry (APACHE) II score were recorded at the first administration of the study drugs [22]. The use of mechanical ventilation and systemic steroids were assessed during the therapeutic period.

### Statistical analysis

All data were presented as mean ± standard deviation and numbers (percentage) for continuous and categorical variables, respectively. Categorical variables were compared using the χ^2^ or Fisher’s exact test. The Kolmogorov–Smirnov test was performed to confirm the normality of data distribution. Differences in continuous variables were analyzed using Student’s t-test. Because the variables did not satisfy normality, the Mann–Whitney U test was performed to analyze the differences in respiratory rate; APACHE II score; creatinine, C-reactive protein, and procalcitonin level, and therapeutic period. Kaplan–Meier analysis was performed to calculate the time-to-composite event curve with a log-rank test.

For univariate and multivariate analyses, a Cox proportional hazard model was used to calculate the adjusted odds ratio (aOR) with 95% confidence interval (CIs). The covariates were selected based on their statistical significance (*p* ≤ 0.05) in the univariate analysis. We added the APACHE II score and body mass index as covariates based on the literature review [23, 24]. All tests of significance were two-sided. *p* values of < 0.05 were considered statistically significant. All analyses were performed using SPSS software (version 24.0; Chicago, IL, USA).

## Results

### Study participant comparison

**Fig. 1:**
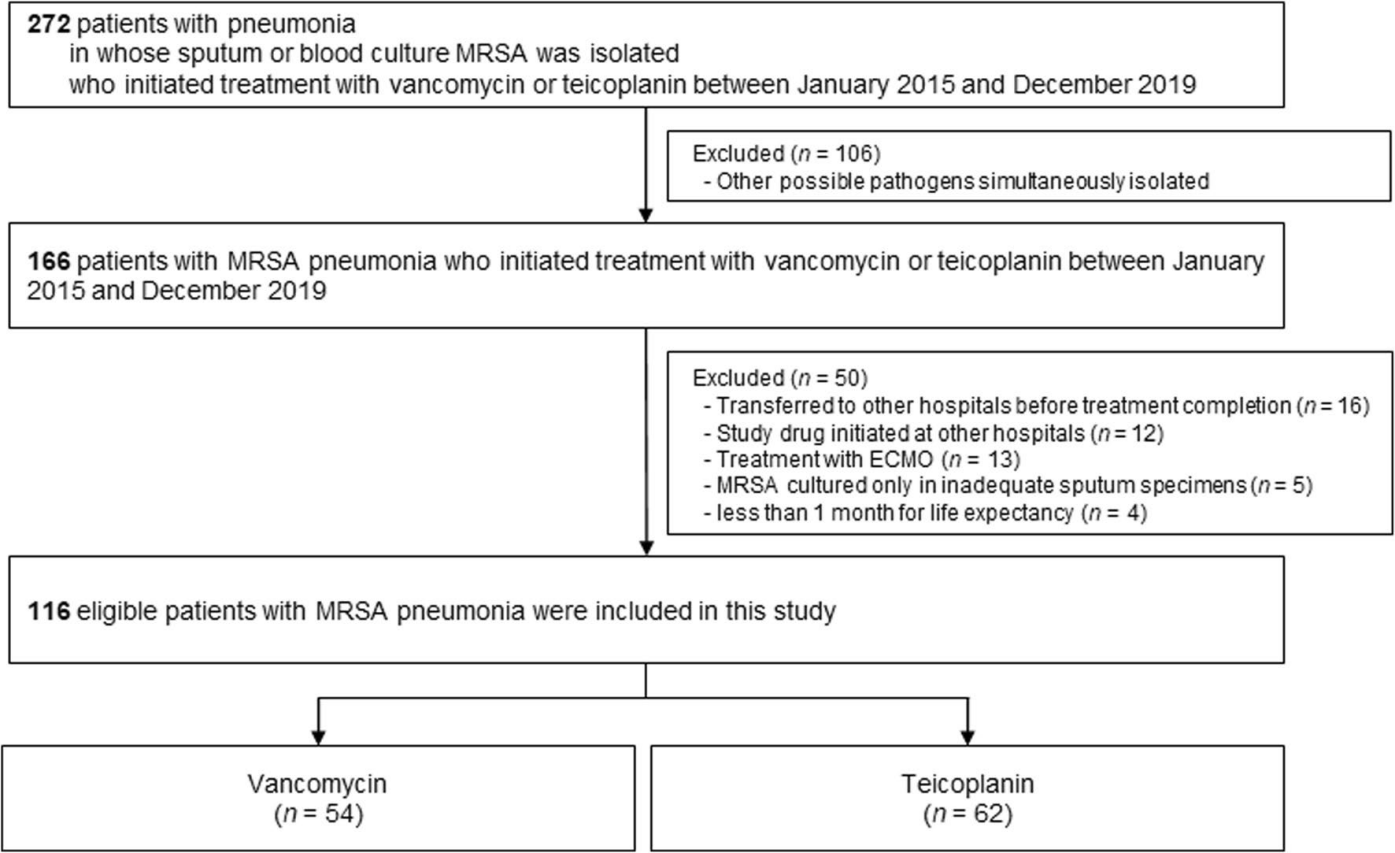
Study flowchart. *ECMO* extracorporeal membrane oxygenation, *MRSA* methicillin-resistant *Staphylococcus aureus*

We screened 272 patients with pneumonia, who had MRSA isolated from their sputum or blood cultures. Among them, MRSA and other potential pathogens were simultaneously isolated in sputum cultures in 106 patients. Because the clinicians did not define MRSA as the only possible pathogen, we excluded these patients. Thus, we reviewed 166 eligible patients with MRSA pneumonia. After review, 116 patients (vancomycin, *n* = 54; teicoplanin, *n* = 62) were included in this study (Fig. 1). The baseline characteristics of the patients are shown in Table 1. Their mean age was 61.4 years, and 79.3% were males. The mean body mass index was 21.3 kg/m^2^. The mean therapeutic duration for MRSA pneumonia was 14.7 days in both groups.

**Table 1:** Baseline characteristics of the patients according to the study groups

Variable	Total (*n* = 116)	Vancomycin (*n* = 54)	Teicoplanin (*n* = 62)	*p* value
Male	92 (79.3%)	39 (72.2%)	53 (85.5%)	0.079
Age (years)	61.4 ± 13.1	67.7 ± 12.9	67.2 ± 13.4	0.842
Body mass index (kg/m^2^)	21.3 ± 4.3	20.5 ± 4.6	22.1 ± 3.9	0.050
Coexisting condition				
DM	33 (28.4%)	14 (25.9%)	19 (30.6%)	0.574
HTN	46 (39.7%)	19 (35.2%)	27 (43.5%)	0.358
Pulmonary disease	38 (32.8%)	11 (20.4%)	27 (43.5%)	0.009
Cardiovascular disease	31 (26.7%)	10 (18.5%)	21 (33.9%)	0.062
Renal disease	12 (10.3%)	6 (11.1%)	6 (9.7%)	0.800
Cerebrovascular disease	31 (26.7%)	21 (38.9%)	10 (16.1%)	0.006
Liver disease^a^	10 (8.6%)	3 (5.6%)	7 (11.3%)	0.334
History of transplantation^a^	10 (8.6%)	9 (16.7%)	1 (1.6%)	0.006
Malignancy				0.288
Solid	33 (28.4%)	14 (25.9%)	19 (30.6%)	
Hematologic	10 (8.6%)	7 (13.0%)	3 (4.8%)	
Recent chemotherapy	26 (22.4%)	11 (20.4%)	15 (24.2%)	0.622
Recent radiotherapy^a^	6 (5.2%)	3 (5.6%)	3 (4.8%)	> 0.999
Neurologic disease	19 (16.4%)	10 (18.5%)	9 (14.5%)	0.561
Type of pneumonia				0.107
CAP	37(31.9%)	17 (31.5%)	20 (32.3%)	
HAP	37 (31.9%)	22 (40.7%)	15 (24.2%)	
VAP	42 (36.2%)	15 (27.8%)	27 (43.5%)	
Bacteremia	20 (17.2%)	10 (18.5%)	10 (16.1%)	0.734
MIC				
Vancomycin				0.569
≤ 0.5	1 (0.9%)	0 (0.0%)	1 (1.6%)	
1	87 (75.0%)	42 (77.8%)	45 (72.6%)	
2	28 (24.1%)	12 (22.2%)	16 (25.8%)	
Teicoplanin^a^				> 0.999
≤ 4	112 (96.6%)	52 (96.3%)	60 (96.8%)	
8	4 (3.4%)	2 (3.7%)	2 (3.2%)	
Initial vital sign				
SBP	121.5 ± 22.6	119.8 ± 21.3	123.0 ± 23.7	0.448
DBP	68.0 ± 13.9	67.1 ± 14.5	68.7 ± 13.4	0.517
Pulse rate	98.9 ± 21.4	99.8 ± 21.6	98.2 ± 21.5	0.699
Body temperature	37.2 ± 0.8	37.2 ± 0.8	37.2 ± 0.8	0.673
Respiratory rate^b^	22.3 ± 5.8	21.9 ± 6.0	22.7 ± 5.7	0.801
Mechanical ventilation	70 (60.3%)	30 (55.6%)	40 (64.5%)	0.325
Systemic steroids	31 (26.7%)	13 (24.1%)	18 (29.0%)	0.547
APACHE II score^b^	13.5 ± 5.3	13.1 ± 5.4	13.9 ± 5.2	0.324
White blood cell counts	11.9 ± 5.7	11.6 ± 5.6	12.1 ± 5.9	0.609
C-reactive protein^b^	11.0 ± 8.6	12.1 ± 7.9	10.1 ± 9.2	0.045
Procalcitonin (*n* = 79) ^b^	5.4 ± 19.1	1.5 ± 2.8	9.4 ± 26.6	0.367
Creatinine^b^	1.2 ± 1.2	1.1 ± 1.2	1.3 ± 1.1	0.008
Vancomycin serum trough levels (µg/mL)				
Day 3 (*n* = 45)	n/a	13.5 ± 8.1	n/a	
Day 6 (*n* = 34)	n/a	20.4 ± 8.3	n/a	
Day 9 (*n* = 29)	n/a	17.3 ± 4.7	n/a	
Teicoplanin regimen				
High-dose regimen	n/a	n/a	15 (24.2%)	
Low-dose regimen	n/a	n/a	47 (75.8%)	
Therapeutic duration, days^b^	14.7 ± 8.2	14.7 ± 9.1	14.7 ± 7.4	0.658

*APACHE II score* Acute Physiology and Chronic Health Enquiry II score, *CAP* community-acquired pneumonia, *DBP* diastolic blood pressure, *DM* diabetes mellitus, *HAP* hospital-acquired pneumonia, *HTN* hypertension, *MIC* minimal inhibition concentration, *SBP* systolic blood pressure, *VAP* ventilator-associated pneumonia

^a^Variables analyzed using Fisher’s exact test

^b^Variables analyzed using the Mann–Whitney U test

The most common type of pneumonia was HAP and VAP in the vancomycin and teicoplanin groups, respectively. No difference was noted between the two groups in terms of the APACHE II score. There was a significant difference between the groups in serum creatinine and C-reactive protein levels at the first administration of the study drugs. Patients with an underlying pulmonary disease were included at a higher proportion in the teicoplanin group. Additionally, patients with cerebrovascular disease and a history of organ transplantation were included at a higher proportion in the vancomycin group.

### Primary and secondary outcomes in both groups

**Fig. 2:**
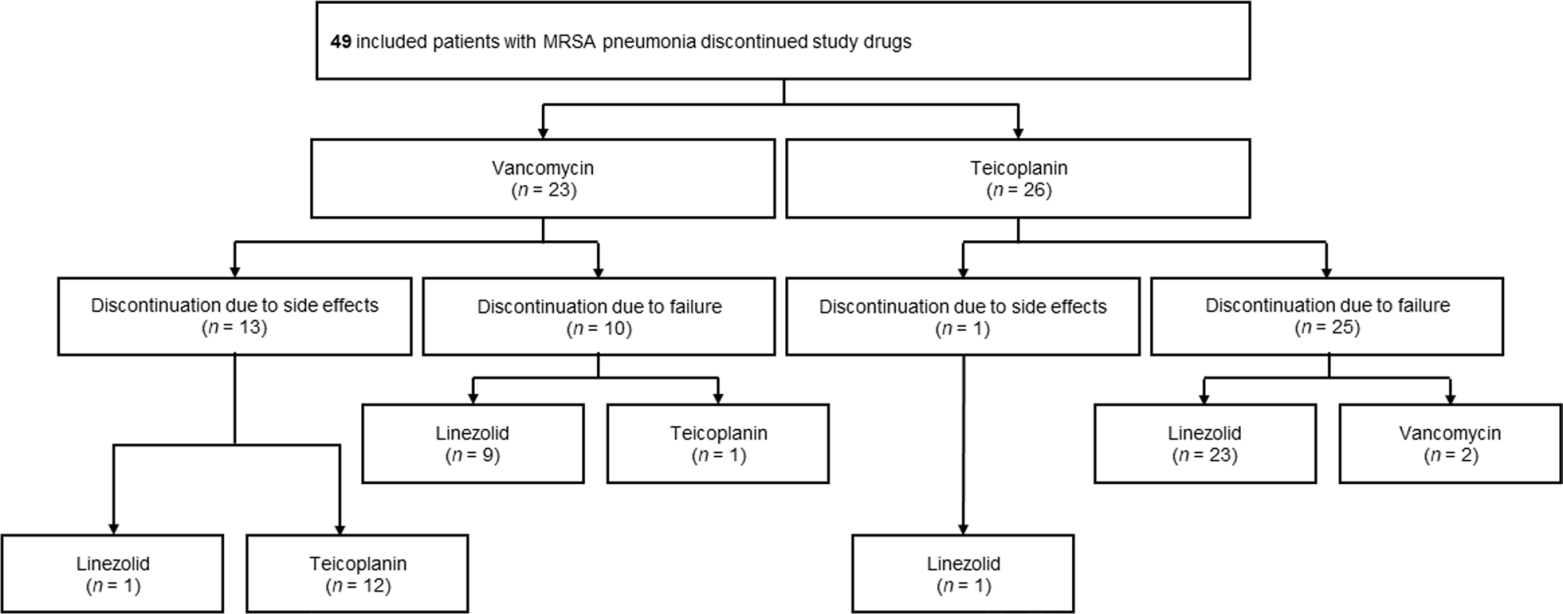
Flowchart showing the changes of study drugs. *MRSA* methicillin-resistant *Staphylococcus aureus*

Table 2 presents the primary and secondary outcomes during the therapeutic period in both groups. The vancomycin group presented lower rates of clinical failure (25.9% vs. 61.3%, *p* < 0.001), change of the study drugs due to treatment failure (22.2% vs. 41.9%, *p* = 0.024), and mortality (3.7% vs. 19.4%, *p* = 0.010) than the teicoplanin group, with statistical significance. In both groups, the drug was changed to linezolid for most patients when the clinicians suspected treatment failure and discontinued the study drugs (Fig. 2). Although no statistical significance was noted, the clinical cure rate of vancomycin was higher than that of teicoplanin (50.0% vs. 37.1%, *p* = 0.162).

**Table 2:** Primary and secondary outcomes in both groups

Outcome	Vancomycin (*n* = 54)	Teicoplanin (*n* = 62)	*p* value
Clinical failure	14 (25.9%)	38 (61.3%)	< 0.001
Discontinuation due to treatment failure	12 (22.2%)	26 (41.9%)	0.024
Death	2 (3.7%)	12 (19.4%)	0.010
Discontinuation due to side effects	13 (24.1%)	1 (1.6%)	< 0.001
Clinical cure	27 (50.0%)	23 (37.1%)	0.162

Additional analysis was conducted for the primary and secondary outcomes after excluding patients in whom the study drugs were changed due to side effects (Additional file 1). In this analysis, vancomycin presented favorable outcomes with statistical significance, except for treatment failure.

We performed subgroup analysis classified by the type of pneumonia (Additional file 2). In the VAP subgroup, clinical cure and failure rate were significantly favorable in the vancomycin group. In the HAP subgroup, the vancomycin group presented a lower clinical failure rate than the teicoplanin group. Although the number of patients in whom the study drugs were discontinued due to side effects was higher in the vancomycin group with all pneumonia types, statistical significance was presented only in the CAP subgroup.

### Univariate and multivariate analysis of primary outcome

**Fig. 3:**
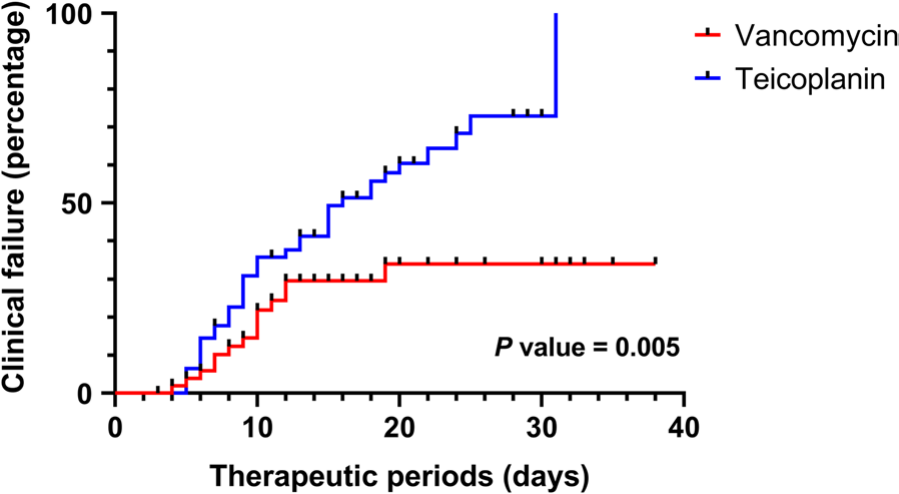
Kaplan–Meier cumulative event rates for the composite event during the therapeutic period

Figure 3 shows the Kaplan–Meier curves of the time to clinical failure events among patients treated with either vancomycin or teicoplanin compared with using the log-rank test. A significant difference in time to clinical failure events was noted between the two groups (*p* = 0.005). The Cox proportional hazard model was used to analyze covariates, which revealed that the hazard ratio for clinical failure events was increased. In the univariate analysis, body mass index, combined cardiovascular disease, transplantation history, and types of pneumonia and antibiotics for MRSA pneumonia were significant factors associated with clinical failure events (Table 3). Multivariate analysis revealed that the use of teicoplanin (aOR, 2.198; 95% CI 1.163–4.154) was a significant predictor of clinical failure compared with vancomycin.

**Table 3:** Univariate and multivariate analysis of clinical failure in the Cox proportional hazard model

Covariate	Composite outcome (*n* = 52)	No composite outcome (*n* = 64)	*p* value	Univariate analysis	Multivariate analysis
				OR (95% CI)	aOR (95% CI)
Male	42 (80.8%)	50 (78.1%)	0.727	1.282 (0.642–2.561)	
Age (years)	67.3 ± 13.9	67.6 ± 12.6	0.899	1.002 (0.980–1.024)	
Body mass index	22.1 ± 4.2	20.7 ± 4.3	0.072	1.044 (0.983–1.110)	1.051 (0.977–1.132)
Pulmonary disease	17 (32.7%)	21 (32.8%)	0.989	0.918 (0.514–1.641)	
Cardiovascular disease	18 (34.6%)	13 (20.3%)	0.083	1.467 (0.828–2.598)	
Renal disease	7 (13.5%)	5 (7.8%)	0.320	1.330 (0.599–2.953)	
Malignancy			0.314		
Solid	18 (34.6%)	15 (23.4%)		1.189 (0.660–2.140)	
Hematologic	3 (5.8%)	7 (10.9%)		0.632 (0.193–2.071)	
Transplantation^a^	1 (1.9%)	9 (14.1%)	0.022	0.193 (0.027–1.396)	0.368 (0.049–2.783)
Liver disease	4 (7.7%)	6 (9.4%)	> 0.999	0.786 (0.283–2.183)	
Cerebrovascular disease	15 (28.8%)	16 (25.0%)	0.642	1.304 (0.715–2.378)	
Diabetes mellitus	13 (25.0%)	20 (31.3%)	0.458	0.700 (0.373–1.313)	
Bacteremia	9 (17.3%)	11 (17.2%)	0.986	0.924 (0.449–1.901)	
APACHE II	14.4 ± 5.5	12.7 ± 5.1	0.088	1.030 (0.983–1.083)	1.024 (0.972–1.078)
CRP	11.7 ± 9.9	10.5 ± 7.5	0.506	1.010 (0.980–1.041)	
Type			0.050		
CAP	11 (21.2%)	26 (40.6%)		Reference	Reference
HAP	17 (32.7%)	20 (31.3%)		1.984 (0.925–4.256)	2.165 (0.996–4.708)
VAP	24 (48.2%)	18 (28.1%)		1.942 (0.950–3.973)	1.496 (0.702–3.189)
Treatment			< 0.001		
Vancomycin	14 (26.9%)	40 (62.5%)		Reference	Reference
Teicoplanin	38 (73.1%)	24 (37.5%)		2.339 (1.259–4.346)	2.198 (1.163–4.154)

The variables in the “composite outcome” and “no composite outcome” groups were compared using the χ^2^ or Fisher’s exact test for categorical variables and the Student’s *t*-test for continuous variables. Additionally, the univariate and multivariate analyses were conducted using the Cox proportional hazard model

*APACHE II score* Acute Physiology and Chronic Health Enquiry II score, *CAP* community-acquired pneumonia, *CI* confidence interval, *CRP* C-reactive protein, *HAP* hospital-acquired pneumonia, *OR* odds ratio, *VAP* ventilator-associated pneumonia

^a^Variables analyzed using Fisher’s exact test

### Side effects of vancomycin and teicoplanin

The rates of drug change due to side effects were higher in the vancomycin group (24.1% vs 1.6%, *p* < 0.001; Table 2). The cause of discontinuation of the study drugs is presented in Table 4. We also investigated the occurrence of adverse effects of the study drugs in the setting of concomitant bacteremia. In the vancomycin group, 2 patients discontinued vancomycin due to adverse effects among 10 patients with bacteremia. Among 44 patients without bacteremia, 11 patients discontinued vancomycin due to adverse effects. In the teicoplanin group, 1 patient discontinued teicoplanin and did not have accompanying bacteremia. Azotemia is the most common side effect of vancomycin, followed by elevated liver function tests. Among 13 patients, the study drug was changed to teicoplanin in 12 patients in the vancomycin group due to adverse drug effects (Fig. 2). After discontinuing vancomycin, the adverse effects among 8 patients improved. However, the remaining 5 patients, 4 with azotemia and 1 with thrombocytopenia, did not improve after vancomycin discontinuation. Only one patient discontinued teicoplanin due to skin rash and shifted to linezolid. After shifting to linezolid, the skin rash resolved.

**Table 4:** Cause of discontinuation of the study drugs in both groups

	Vancomycin (*n* = 54)	Teicoplanin (*n* = 62)
Clinical failure	10 (18.5%)	25 (40.3%)
Azotemia	8 (14.8%)	0 (0.0%)
Elevated liver function test	2 (3.7%)	0 (0.0%)
Skin rash	1 (1.9%)	1 (1.6%)
Eosinophilia	1 (1.9%)	0 (0.0%)
Thrombocytopenia	1 (1.9%)	0 (0.0%)

## Discussion

In this study, vancomycin presented favorable efficacy outcomes compared with teicoplanin, although the rate of discontinuation of the study drugs due to side effects was higher in the vancomycin group. Treatment outcome, including mortality and discontinuation of the study drugs due to treatment failure, was considered a clinical failure of the study drugs. Although concerns regarding the possibility of overestimation of the clinical failure rate were noted, there were significant differences in the mortality and discontinuation rates due to treatment failure between vancomycin and teicoplanin. Furthermore, the clinical cure rate was favorable in the vancomycin group, although no statistical significance was noted. Thus, the clinical cure rate may be insufficient to retrospectively evaluate the efficacy and safety of vancomycin and teicoplanin with a relatively small number of patients because linezolid could be actively considered as an alternative for patients with MRSA pneumonia after Wunderink’s study [20, 25]. Furthermore, the clinicians in the present study changed the study drugs to linezolid in 27.6% (32/116) of patients because they suspected treatment failure of the study drugs in nine patients in the vancomycin group and 23 patients in the teicoplanin group. More side effects were noted, leading to the discontinuation of the study drugs in the vancomycin group compared with that in the teicoplanin group. We selected clinical failure as the primary outcome owing to treatment failure because these changes could lead to the underestimation of treatment efficacy. Moreover, the treatment efficacy of vancomycin and teicoplanin were analyzed after excluding patients with adverse effects, as shown in Additional file 1. To the best of our knowledge, this is the first study to show that the outcomes of vancomycin are more favorable than those of teicoplanin in patients with MRSA pneumonia.

Glycopeptides are reported to present relatively lower lung penetration. Although teicoplanin presented a higher lung penetration ratio than vancomycin in previous studies, no clear evidence was presented [13, 26–30]. The penetration of various antibiotics into the lungs of patients with HAP and VAP was found to be variable [31–33]. Furthermore, the protein-binding rate of teicoplanin was variable in critically ill patients [34]. Therefore, clinicians could not agree that patients with MRSA pneumonia individually took adequate teicoplanin doses. Physicians could individually adjust the vancomycin dose based on therapeutic drug monitoring levels. The efficacy of vancomycin and teicoplanin depends on the trough drug concentration [35]. Therefore, variability possibly affected the efficacy of teicoplanin. Recently, the importance of therapeutic drug monitoring of teicoplanin has been increasingly emphasized [35].

Another possible cause of unfavorable teicoplanin outcomes in this study was that more than 50% of the patients in the teicoplanin group were prescribed a low-dose regimen. Mimoz et al. indicated that patients with VAP required a high-dose teicoplanin regimen to maintain adequate trough levels in the lungs [28]. However, there was no difference in the clinical failure rate (60.0% vs. 61.7%, *p* = 0.906), clinical cure rate (40.0% vs. 36.2%, *p* = 0.789), and adverse event rate (0.0% vs. 2.1%, *p* > 0.999) between the high-dose and low-dose groups (Additional file 3). Even in multivariate analysis for clinical failure, there was no significant difference between the two groups, although high-dose teicoplanin presented a lower aOR. Another possible cause was that more patients with underlying respiratory or cardiovascular disease were enrolled in the teicoplanin group. Although previous medical history did not affect the composite events in the Cox proportional hazard model, further studies are needed to evaluate the effects of teicoplanin dose and these covariates because a relatively small number of patients were included in this study.

In this study, more patients in the vancomycin group had to discontinue the study drugs due to adverse effects. Azotemia was the most common adverse effect in this study, which is similar to the results of other studies [8, 36]. The nephrotoxicity of vancomycin is a well-known side effect. Various risk factors are associated with vancomycin-induced nephrotoxicity, including vancomycin trough level of ≥ 16.2 µg/mL [37, 38]. Furthermore, nosocomial pneumonia is regarded as a possible risk factor for vancomycin-induced nephrotoxicity because MRSA pneumonia usually requires a higher dose of vancomycin because of poor lung penetration and lower susceptibility to antibiotics if they are associated with hospital-acquired infection [38, 39]. The mean vancomycin trough levels were > 16.2 µg/mL at days 6 and 9 during the therapeutic period in this study because most of the clinicians attempted to maintain the trough level between 15 and 20 µg/mL. Relatively high concentrations may affect azotemia. Conversely, only one case of adverse effect was noted in the teicoplanin group. Bacteremia is also considered an important cause of nephrotoxicity as it is a sign of more serious disease. However, no obvious difference was found between the groups with and without bacteremia. However, no obvious difference was found between the groups with and without bacteremia. Thrombocytopenia is a well-known complication of teicoplanin administration [40]. Because vancomycin presented unfavorable outcomes owing to side effects and poor prognosis was reported in pneumonia patients with acute kidney injury in a previous study, teicoplanin could be considered as an alternative, especially in patients with risk factors associated with vancomycin-induced nephrotoxicity [41]. In our study, among the 13 patients who discontinued vancomycin due to adverse effects, vancomycin was replaced with teicoplanin in 12 patients. The rate of each side effect was relatively lower than that reported in other studies because only adverse effects leading to the discontinuation of study drugs were included [36, 40, 42].

This study had several limitations. First, the study was conducted at a single referral center using a retrospective design with relatively small populations. Additionally, patients for whom vancomycin or teicoplanin was initiated at other hospitals or who were transferred to other facilities were excluded, which might have led to selection bias. Further randomized controlled studies with a large number of patients with MRSA pneumonia would be needed to clarify the results of this study. Second, MRSA pneumonia was defined to be based on the physicians’ judgments. Although the medical records to confirm the clinical diagnosis and exclude patients with other possible causative pathogens were reviewed, there were possibilities indicating that pneumonia was caused by microorganisms other than MRSA. Third, the discontinuation of the study drugs due to adverse events as outcomes independent of clinical failure and clinical cure were analyzed. As discontinuation of the study drugs due to side effects occurred more in the vancomycin group, the clinical failure rate could be overestimated, which makes it difficult to apply our results to real clinical practice. If physicians could identify patients at high risk for vancomycin-induced adverse effects, our results could effectively help physicians in real practice. Finally, the two-dose teicoplanin regimen was included in this study. Thus, the dose effects for the efficacy of teicoplanin could not be confirmed. Further studies may be needed to evaluate each dose regimen of teicoplanin and vancomycin.

## Conclusions

In this study, patients treated with teicoplanin for MRSA pneumonia presented a higher clinical failure rate than those treated with vancomycin group. Other treatment outcomes, including mortality, treatment failure, and clinical cure, were more favorable in the vancomycin group than in the teicoplanin group. However, more side effects, which led to the change to other antibiotics for continuous management, were noted in the vancomycin group. Although further prospective studies with a large number of patients with MRSA pneumonia are needed to clarify the results of this study, clinicians might need to consider the efficacy and safety profile of vancomycin and teicoplanin for MRSA pneumonia.

## Supplementary Information

**Additional file 1. **Primary and secondary outcomes in both groups after excluding patients in whom the study drug was changed because of side effects.

**Additional file 2. **Primary and secondary outcomes of both groups according to the pneumonia type. *CAP* community-acquired pneumonia, *HAP* hospital-acquired pneumonia, *VAP* ventilator-associated pneumonia.

**Additional file 3. **Primary and secondary outcomes of the low-dose and high-dose teicoplanin groups. We performed the subgroup analysis between the low-dose and high-dose teicoplanin groups. There was no difference of treatment outcome between the two groups. Additionally, we performed univariate and multivariate analysis with Cox proportional hazard model. All covariates included in Table 3 were included. High-dose teicoplanin presented an odds ratio of 0.649 (95% confidence interval 0.295–1.429, *p* = 0.283) in univariate analysis and 0.641 (95% confidence interval 0.243–1.690, *p* = 0.369) in multivariate analysis.

## Data Availability

The datasets used and/or analysed during the current study are available from the corresponding author on reasonable request.

## Abbreviations

aOR: Adjusted odds ratio
APACHE II: Acute Physiology and Chronic Health Enquiry II
CAP: Community-acquired pneumonia
CI: Confidence interval
HAP: Hospital-acquired pneumonia
MRSA: Methicillin-resistant *Staphylococcus aureus*
VAP: Ventilator-associated pneumonia
