# Supplementary material for: Comparison of mortality and clinical failure rates between vancomycin and teicoplanin in patients with methicillin-resistant Staphylococcus aureus pneumonia
Source: BMC Infect Dis. 2022 Jul 7;22:600. doi: 10.1186/s12879-022-07549-2 (PMC9264637; doi:10.1186/s12879-022-07549-2)
Supplement: Supplementary file 1 — Additional file 1. Primary and secondary outcomes in both groups after excluding patients in whom the study drug was changed because of side effects. [file 12879_2022_7549_MOESM1_ESM.pdf]

Additional file 1. Primary and secondary outcomes in both groups after excluding patients in whom the study drug was changed because of side effects.

Outcome	Vancomycin (n = 41)	Teicoplanin (n = 61)	P value
Clinical failure	14 (34.1%)	38 (62.3%)	0.008
Treatment failure	12 (29.3%)	26 (42.6%)	0.212
Mortality	2 (4.9%)	12 (19.7%)	0.041
Clinical cure	27 (65.9%)	23 (37.7%)	0.008
