# Supplementary material for: Comparison of mortality and clinical failure rates between vancomycin and teicoplanin in patients with methicillin-resistant Staphylococcus aureus pneumonia
Source: BMC Infect Dis. 2022 Jul 7;22:600. doi: 10.1186/s12879-022-07549-2 (PMC9264637; doi:10.1186/s12879-022-07549-2)
Supplement: Supplementary file 2 — Additional file 2. Primary and secondary outcomes of both groups according to the pneumonia type. CAP community-acquired pneumonia, HAP hospital-acquired pneumonia, VAP ventilator-associated pneumonia. [file 12879_2022_7549_MOESM2_ESM.pdf]

Additional file 2. Primary and secondary outcomes of both groups according to the pneumonia type

	Total	Vancomycin	Teicoplanin	*P* value
VAP	n = 42	n= 15	n= 27	
Clinical cure	16 (38.1%)	9 (60.0%)	7 (25.9%)	0.029
Clinical failure	24 (57.1%)	4 (26.7%)	20 (74.1%)	0.003
Treatment failure	20 (47.6%)	4 (26.7%)	16 (59.3%)	0.043
Death*	4 (9.5%)	0 (0.0%)	4 (14.8%)	0.279
Discontinuation due to side effects*	2 (4.8%)	2 (13.3%)	0 (0.0%)	0.122
HAP	N = 37	N = 22	N = 15	
Clinical cure	13 (35.1%)	9 (40.9%)	4 (26.7%)	0.373
Clinical failure	17 (45.9%)	7 (31.8%)	10 (66.7%)	0.037
Treatment failure*	11 (29.7%)	5 (22.7%)	6 (40.0%)	0.295
Death*	6 (16.2%)	2 (9.1%)	4 (26.7%)	0.198
Discontinuation due to side effects*	7 (18.9%)	6 (27.3%)	1 (6.7%)	0.204
CAP	N = 37	N = 17	N = 20	
Clinical cure	21 (56.8%)	9 (52.9%)	12 (60.0%)	0.666
Clinical failure	11 (29.7%)	3 (17.6%)	8 (40.0%)	0.138
Treatment failure*	7 (18.9%)	3 (17.6%)	4 (20.0%)	>0.999
Death*	4 (10.8%)	0 (0.0%)	4 (20.0%)	0.109
Discontinuation due to side effects*	5 (13.5%)	5 (29.4%)	0 (0.0%)	0.014

CAP community-acquired pneumonia, *HAP* hospital-acquired pneumonia, *VAP* ventilator-associated pneumonia
