# Supplementary material for: Comparison of mortality and clinical failure rates between vancomycin and teicoplanin in patients with methicillin-resistant Staphylococcus aureus pneumonia
Source: BMC Infect Dis. 2022 Jul 7;22:600. doi: 10.1186/s12879-022-07549-2 (PMC9264637; doi:10.1186/s12879-022-07549-2)
Supplement: Supplementary file 3 — Additional file 3. Primary and secondary outcomes of the low-dose and high-dose teicoplanin groups. We performed the subgroup analysis between the low-dose and high-dose teicoplanin groups. There was no difference of treatment outcome between the two groups. Additionally, we performed univariate and multivariate analysis with Cox proportional hazard model. All covariates included in Table 3 were included. High-dose teicoplanin presented an odds ratio of 0.649 (95% confidence interval 0.295–1.429, p = 0.283) in univariate analysis and 0.641 (95% confidence interval 0.243–1.690, p = 0.369) in multivariate analysis. [file 12879_2022_7549_MOESM3_ESM.pdf]

Additional file 3. Primary and secondary outcomes of the low-dose and high-dose teicoplanin groups

Outcome	Total (n = 62)	Low-dose Teicoplanin (*n* = 47)	High-dose Teicoplanin (*n* = 15)	*P* value
Clinical cure	23 (37.1%)	17 (36.2%)	6 (40.0%)	0.789
Clinical failure	38 (61.3%)	29 (61.7%)	9 (60.0%)	0906
Treatment failure	26 (41.9%)	22 (46.8%)	4 (26.7%)	0.169
Death*	12 (19.4%)	7 (14.9%)	5 (33.3%)	0.142
Discontinuation due to side effects*	1 (1.6%)	1 (2.1%)	0 (0.0%)	>0.999

We performed the subgroup analysis between the low-dose and high-dose teicoplanin groups. There was no difference of treatment outcome between the two groups. Additionally, we performed univariate and multivariate analysis with Cox proportional hazard model. All covariates included in Table 3 were included. High-dose teicoplanin presented an odds ratio of 0.649 (95% confidence interval 0.295–1.429, $P = 0.283$) in univariate analysis and 0.641 (95% confidence interval 0.243–1.690, $P = 0.369$) in multivariate analysis.
